# Effect of prenatal exposure to stress and extremely low-frequency electromagnetic field on hippocampal and serum BDNF levels in male adult rat offspring 

**DOI:** 10.22038/IJBMS.2024.75459.16357

**Published:** 2024

**Authors:** Hajar Abkhezr, Shirin Babri, Mahsa Farid-Habibi, Fereshteh Farajdokht, Saeed Sadigh-Eteghad, Gisou Mohaddes

**Affiliations:** 1 Tabriz University of Medical Sciences, Tabriz, Iran; 2 Shahid Beheshti University of Medical Sciences, Tehran, Iran; 3 Neurosciences Research Center, Tabriz University of Medical Sciences, Tabriz, Iran; 4 Department of Biomedical Education, California Health Sciences University, College of Osteopathic Medicine, Clovis, CA, USA

**Keywords:** Brain-derived - neurotrophic factor, Corticosterone, ELF-EMF, Prenatal stress, Spatial memory

## Abstract

**Objective(s):**

Prenatal stress (PS) can adversely affect cognitive and psychological functions in the offspring. This study aimed to determine the effect of PS and extremely low-frequency electromagnetic field (ELF-EMF) on spatial memory, serum corticosterone, brain-derived neurotrophic factor (BDNF) concentrations, and hippocampal BDNF levels in adult male offspring.

**Materials and Methods:**

Female Wistar rats were randomly divided into four groups (n=6): Control, Stress, ELF-EMF (exposure to ELF-EMF), and S+EMF (simultaneous exposure to stress and the ELF-EMF) groups. Animals received interven-tions for 21 days before and 21 days during pregnancy (a total of 42 days). On the offspring’s 90^th^ postnatal day (PND), spatial memory was tested using Morris Water Maze, serum Corticosterone and BDNF levels were measured by the ELISA method, and hippocampal BDNF levels were measured by Western blotting.

**Results:**

PS did not affect spatial memory in the adult male offspring; however, it significantly (*P*<0.05) increased se-rum corticosterone levels compared to the control and EMF groups. Simultaneous induction of stress with ELF-EMF disrupted the memory acquisition phase. Serum and hippocampal BDNF levels increased signifi-cantly (*P*<0.05) in the EMF group compared to the stress group.

**Conclusion:**

Based on our findings, PS can increase serum corticosterone levels without affecting spatial memory. Howev-er, induction of ELF-EMF with stress has a destructive effect on spatial memory with no change in the corti-costerone levels. Compared to stress, prenatal exposure to ELF-EMF increases serum and hippocampal BDNF levels. Further studies are needed to determine the underlying mechanisms of these findings.

## Introduction

Pregnancy is a sensitive period with brain developments and neurobehavioral changes in offspring (1). The organism’s development is subjected to complex environmental influences during the perinatal period. Deleterious life events during pregnancy induce neurobiological and behavioral defects in offspring (2-6). In recent years, there has been growing concern about the potential impact of prenatal stress (PS) exposure on the development of the adult brain. Several studies have been performed on PS, but the results are contradictory. PS has been shown to adversely affect cognitive and psychological functions in the offspring (7). PS by prolonged maternal restraint was reported to slow the acquisition of spatial learning in the Morris water maze. This was associated with reduced hippocampal neurogenesis in the offspring (8). On the other hand, shorter periods of restraint or other forms of maternal stress did not impair the acquisition of spatial learning in males or females (9). Interestingly, mild gestational stress improved learning in male offspring (10, 11). A few studies also show the impact of stress before pregnancy on the memory of offspring (10). 

Brain-derived neurotrophic factor (BDNF) is a neurotrophin family member that plays a vital role in promoting neuronal survival, axonal guidance (11), synaptic transmission, and plasticity within the hippocampus (11). Maternal stress influences the development of hippocampal functions in the offspring by reducing BDNF (12). It may be inferred that a decrease in the expression of neurotrophins in the offspring of pregestational stress could lead to impairment in neurotransmitter secretion (13). PS may also contribute to anxiety later in life in female offspring (14). In addition, PS has been shown to increase glucocorticoid levels in the hippocampus of the offspring (14). Recent findings demonstrate that the interplay between glucocorticoids and BDNF may have a role in adaptive responses to stress. Glucocorticoid receptor activation was shown to recruit BDNF signaling pathways to consolidate contextual fear memories (15). The interplay between glucocorticoids and BDNF, with the influence of other molecules (16), may work as a facilitator of plasticity in adaptive stress response (17). However, human and animal studies have reported an inverse relationship between glucocorticoids and BDNF levels (18). 

Today, all humans, including pregnant women, are continuously exposed to different levels of electromagnetic fields (EMFs) from various sources, such as mobile phones, mobile base stations, and Wi-Fi. EMFs with frequencies between 3 Hz and 300 Hz are generally known as ELF-EMF fields (19). There are rising concerns over the biological effects of extremely low ELF-EMF. Some studies show prenatal exposure to ELF-EMF increases the risk of fetal developmental disorders and congenital malformations, possibly due to increased oxidative stress reactions and DNA damage (20). Few studies have examined the effects of prenatal exposure to ELF-EMF on spatial memory and brain BDNF levels. Daily treatment with pulsed ELF-EMF significantly diminished the pro-inflammatory response by reducing microglia and astroglia activation and increasing BDNF expression in mice (21). Another study showed that a pulsed electromagnetic field at 50 Hz and 1 mT increased BDNF expression in dorsal root ganglion neurons (22). However, recent research on prenatal exposure to ELF-EMF indicated a decrease in BDNF expression in the prefrontal cortex of female offspring. Another recent study mentioned that exposure to EMF during the brain development period changed the levels of synaptic proteins, which resulted in behavioral alterations in a gender-specific way (23). The laboratory results regarding the effects of ELF-EMFs on human health are controversial and no definite conclusion can be drawn (24). 

Both PS and EMF exposure have been individually studied for their potential effects on fetal development and neurodevelopmental outcomes. However, there is a lack of comprehensive research examining the combined impact of these two factors on the adult brain. In the present study, we examined the effect of PS and ELF-EMF exposure on spatial memory, serum corticosterone and BDNF concentrations, and hippocampal BDNF levels of adult male offspring.

## Materials and Methods

***Animals and experimental groups***

Twenty-four female and twelve male adult Wistar rats (3 months, 200–250 g) were purchased from the Animal Center of Tabriz University of Medical Science. Animals were kept in groups of six in Plexiglas cages and housed in a room with controlled temperature (23 ±0.5 °C) under 12 hr light/dark cycles (lights on 08:00–20:00 hr). They had free water and food access, except during experimental procedures. The research protocol of this study was approved by the Animal Experimentation Ethics Committee of Tabriz University of Medical Sciences (IR.TBZMED.VCR.REC.1397.232). 

**Figure 1:**
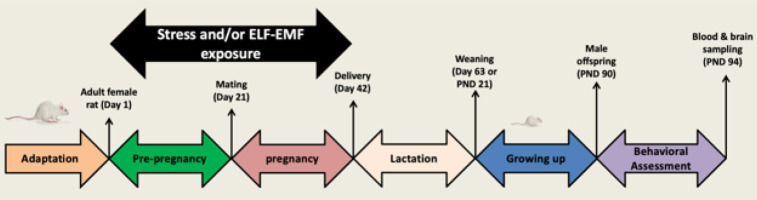
Timeline of the interventions and behavioral tests in female rats and their offspring

Female rats were randomly divided into four experimental groups (n=6): Control (C) group (exposure to off ELF-EMF and without exposure to stressful stimuli); Stress (S) group (exposure to stressful stimuli); Extremely low-frequency electromagnetic field (EMF) group (exposure to ELF-EMF); S+EMF group (simultaneous exposure to stress and ELF-EMF). Animals received interventions before and during pregnancy for a total of 42 days, 21 days before mating, and 21 days during pregnancy. Afterward, two female and one male rat were placed in a cage to mate. After checking and confirming the vaginal plaque, male and female rats were separated. Then, during 21 days of pregnancy, rats in each experimental group were exposed to ELF-EMF and/or stress. Each pregnant rat was transferred to a separate cage during the last week of pregnancy. On the PND 21, male offspring were separated from their mothers. On the 90^th^ PND, male offspring were evaluated for BDNF levels of the hippocampus and serum BDNF and corticosterone concentrations (Figure 1).

***Chronic mild stress***

The CMS protocol used in our study was similar to that of Willner and colleagues, with slight modifications. In brief, rats were subjected to a 45° cage tilt, stroboscopic light, intermittent white noise (80 dB), soiling of cages, food and water deprivation, water deprivation followed by exposure to an empty water bottle, food deprivation followed by restricted food (0.5 g of food pellets), paired housing and reversal of light/dark cycle. These stressors were applied for one week and repeated over three weeks, and each week of the stress regime consisted of eight different stress situations, such as two periods of stroboscopic illumination (300 flashes/min), one period of soil bedding, two periods of white noise (80 dB), two periods of 45° cage tilt, one period of paired housing, periods of food and/or water deprivation where water deprivation was followed by exposure to an empty water bottle. Food deprivation was followed by restricted food (Table 1) (25).

***The electromagnetic field***

**Figure 2:**
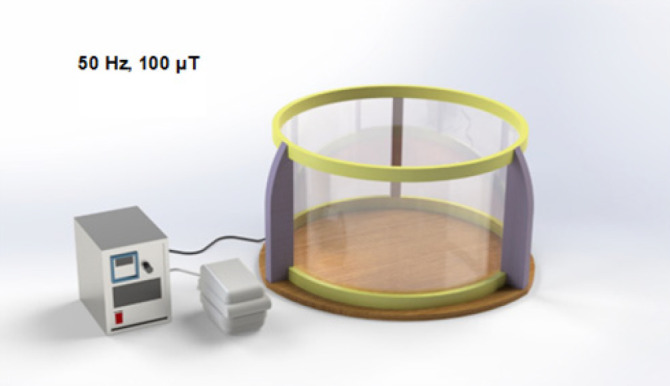
Electromagnetic field exposure system for the induction of magnetic field in female rats

ELF-EMF was produced by an apparatus made according to Helmholtz’s design. The electromagnetic field generator comprised two coaxially circled coils (30 cm in radius, each with 154 turns) separated by a 30 cm distance. The coil carrier ring was made of wood without any metal and was placed at a fixed distance between them using a wooden tripod located on a wooden board. No metal piece was used to produce the electromagnetic field in this system. The uniform electric field was built in the space between the two rings (radius= 30 cm and height= 30 cm). In this study, electromagnetic field intensity was 100 µT with a frequency of 50 Hz in the 4180 triple-axis Gauss meters. Animals were located inside the coil system in the cage that had the same size used for the housing of the rat (4h/day: 10 a.m. to 2 p.m. for 21days before mating and 21 days during pregnancy), and electromagnetic intensity was measured by a digital Tesla Meter (Lutron 828, Taipei, Taiwan) before exposure in a different space of the device to confirm its harmonic distribution. Also, a stabilizer was used to stabilize the voltage signal field (Figure 2) (26).

***Morris water maze***

The spatial memory of offspring was assessed using the Morris Water Maze (MWM) task. As previously mentioned, a two-day protocol was followed to train and test the rats in the water maze (27, 28). In short, the one training session on the first day comprised eight trials split into two blocks that were separated by five minutes each. Blocks 1 and 2’s escape latency and distance traveled parameters were computed and examined. A probe test was conducted on the second day, which involved free swimming without a platform for 60 sec. Both the distance traveled and the swimming time in the target quadrant were recorded. Five minutes after the probe test ended, the rats’ ability to reach a visible platform was tested to gauge their motivation and sensorimotor coordination. Video tracking software EthoVision XT, which is fully automated, was used to record and analyze all of the data. 

***Tissue and blood sampling***

To obtain blood and brain tissues, animals were deeply anesthetized with ketamine (90 mg/kg) and xylazine (10 mg/kg) (22). Blood samples were taken from the heart, and animals were decapitated. Brain tissues were immediately obtained, and bilateral hippocampi were excised. Four hippocampal tissues from each group were randomly selected, quickly frozen in liquid nitrogen, and kept at −80 °C for the BDNF western blotting study. Blood samples were centrifuged at 3000 rpm for 15 min to obtain serum. Serum samples were kept in a −80 °C freezer to measure serum corticosterone and BDNF levels. All samples were kept coded and blinded during measurements in the laboratory.

***Western blotting***

Western blotting was performed according to the methods previously described (23). Briefly, hippocampal tissues were homogenized by a tissue homogenizer in ice-cold RIPA lysis buffer (50 mM Tris−HCl, pH 8.0, 0.1% sodium dodecyl sulfate, 150 mM sodium chloride, 0.5% sodium deoxycholate, and 1.0% NP-40) containing antiprotease cocktail and then were centrifuged at 12,000 × g for 10 min at 4 °C. The supernatant was collected, and its protein concentration was measured by a protein-determining protein kit (Bio-Rad, USA). A mixture (1:1) of the sample with 2X loading buffer was made and then boiled for 5 min and then separated by electrophoresis on 10% SDS-polyacrylamide gels and transferred to a methanol-preactivated polyvinylidene fluoride (PVDF) membrane. The membrane was blocked within bovine serum albumin (BSA) 1% in phosphate-buffered saline (PBS) plus 0.1% Tween 20 for two hours with a gentle shake. Subsequently, the membrane was incubated with primary antibodies (purchased from SANTA CRUS) against BDNF (1:500) and β-actin at 4 °C overnight. Then, the membrane was washed with PBS and incubated with horseradish peroxidase-conjugated (HRP)-labeled secondary antibody. To acknowledge even protein loading, stripping of blots with β-actin antibody (1:300) was performed. The density of each protein and β-actin reference band was quantified using Image J software, and all protein bands were normalized against the β-actin protein (29).

***Statistical analysis***

The data were described as mean ± SEM, and *P*<0.05 was considered statistically significant. The data were analyzed using Graph Pad Prism 6. A paired t-test was used to compare the parameters of traveled distance and escape latency of spatial memory in blocks 1 and 2. One-way ANOVA was used to evaluate the parameters of traveled distance, time spent in the probe test’s target quadrant, serum corticosterone and BDNF levels, and hippocampal BDNF expression between groups. Tukey’s *post hoc* test was used to examine the significance of these differences.

## Results

***Effects of prenatal stress and ELF-EMF exposure on the brain and body weights of adult male offspring***

No significant differences in the body and brain weight of male offspring of mothers exposed to PS and/or ELF-EMF were observed between the study groups (Table 2).

***Effects of prenatal stress and ELF-EMF exposure on the spatial memory of adult male offspring in an MWM task***

**Figure 3:**
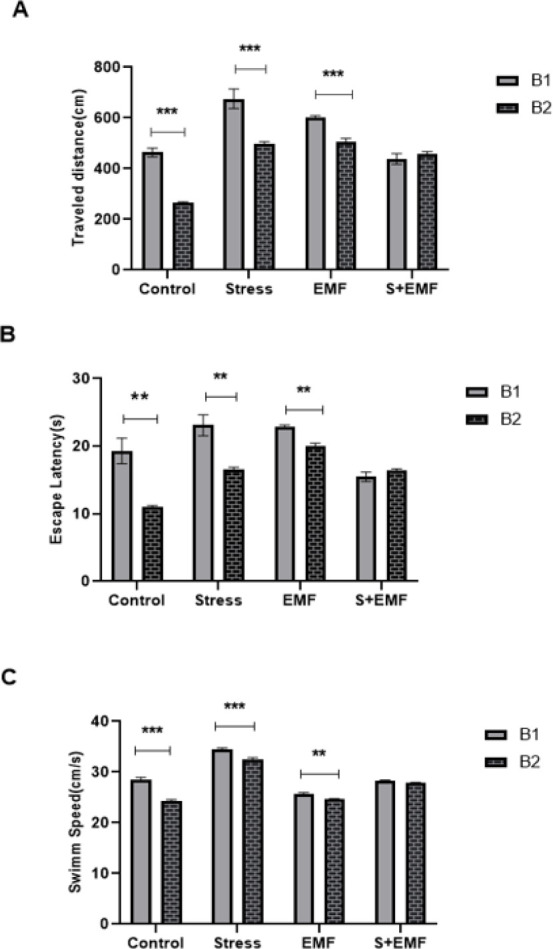
Effects of prenatal stress and ELF-EMF exposure on traveled distance (A), escape latency (B), and swimming speed (C) to find the target platform in the MWM of male adult rat offspring

Three parameters, namely, traveled distance and escape latency to reach the hidden platform and swim speed, were measured in an MWM task. Based on the results of the paired t-test, the traveled distance in block 2 was significantly decreased in the Control (*P*<0.001), Stress (*P*<0.001), and EMF (*P*<0.001) groups compared to block 1. The traveled distance in block 2 was not significantly different from block 1 in the Stress + EMF group, which might indicate a disturbance in the acquisition phase of the memory process (Figure 3A). Escape latency time was also significantly lower in block 2 compared to block 1 in the Control (*P*<0.01), Stress (*P*<0.01), and EMF (*P*<0.01) groups. This parameter in block 2 was not significantly different from block 1 in the Stress + EMF group, which indicates impairment of the memory acquisition phase in this group (Figure 3B). In addition, the swim speed parameter in block 2 was significantly lower than block 1 in the Control (*P*<0.001), Stress (*P*<0.001), and EMF (*P*<0.01) groups. There was no significant difference in the swim speed of blocks in the stress + EMF group (Figure 3C). 

**Figure 4:**
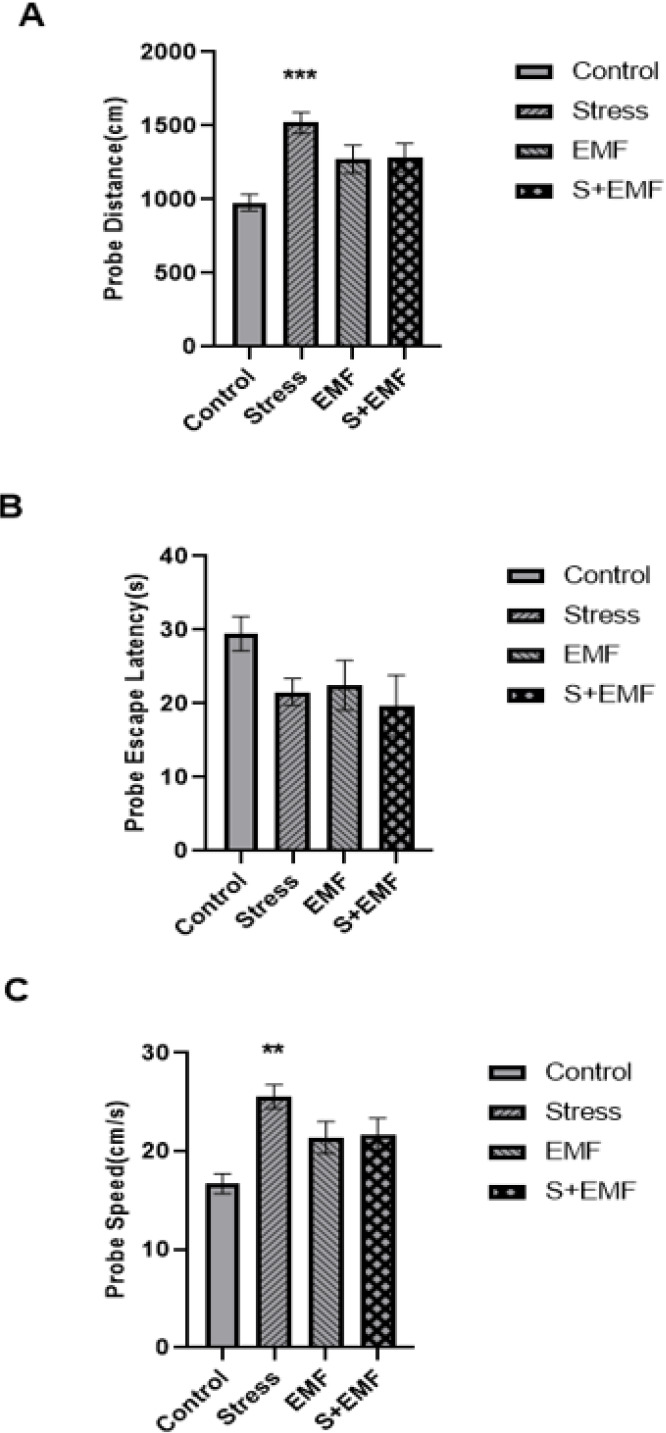
Effects of prenatal stress and ELF-EMF exposure on traveled distance (A), time spent (B), and swim speed (C) in the target quadrant in the MWM of male adult rat offspring

The one-way analysis of variance of the data and Tukey’s *post hoc* test in the traveled distance of probe test in the target quadrant showed a significant difference between the control and stress groups. This parameter was significantly higher (*P*<0.001) in the Stress group compared to the Control group (Figure 4A). No significant difference was observed between the groups in the escape latency in the target quadrant. The absence of significant differences in these two parameters between other groups and the Control group indicates no change (improvement or impairment) of spatial memory in the offspring (Figure 4B). In addition, the swimming speed significantly increased (*P*<0.01) in the Stress group compared to the Control group. A comparison of this parameter did not show a significant difference among the other groups (Figure 4C).

***Impact of prenatal stress and ELF-EMF exposure on serum corticosterone and BDNF levels of adult male offspring***

**Figure 5:**
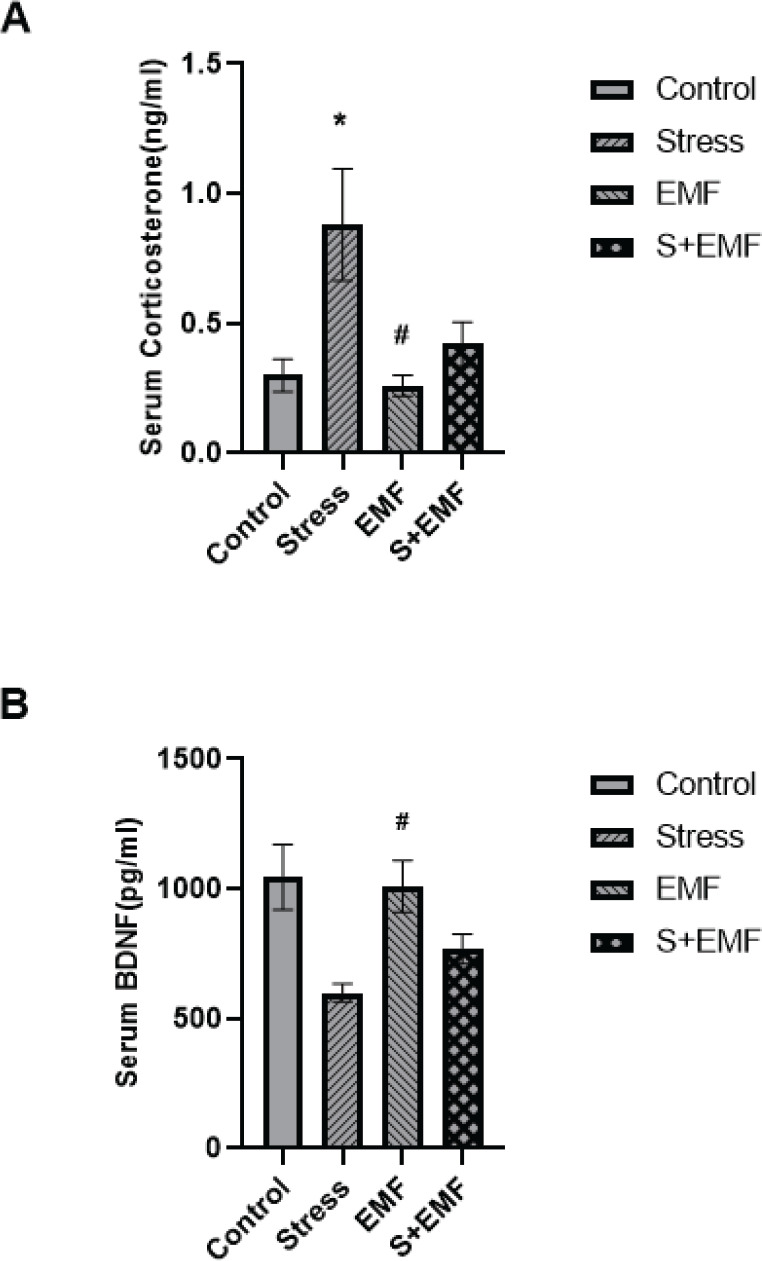
Effects of prenatal stress and ELF-EMF exposure on serum corticosterone (A) and BDNF (B) levels in male adult rat offspring

Serum corticosterone and BDNF levels were measured in the male offspring of mothers exposed to stress and/or ELF-EMF before and during pregnancy. *Post hoc* analysis indicated that maternal stress exposure significantly (*P*<0.05) increased serum corticosterone levels of the offspring compared to the control group. Besides, although it was insignificant, EMF exposure in the stress-subjected animals decreased serum corticosterone levels compared to the stress group. Moreover, serum corticosterone levels were significantly (*P*<0.05) higher in the stress group when compared to the EMF group (Figure 5A). 

*Post hoc* analysis indicated that serum BDNF levels in the EMF group were significantly (*P*<0.05) higher than in the stress group. However, maternal concomitant exposure to stress and EMF did not significantly change male offspring serum BDNF levels compared to the stress and EMF groups. Besides, serum BDNF levels showed an insignificant decrease in the offspring of stress-subjected animals (Figure5B). 

***Effects of prenatal stress and ELF-EMF exposure on the hippocampal BDNF levels***

**Figure 6:**
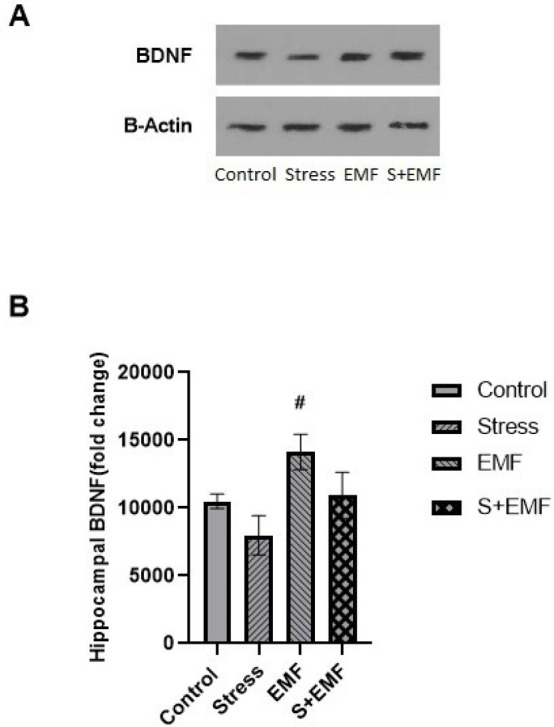
Effects of prenatal stress and ELF-EMF exposure on hippocampal BDNF of male adult rat offspring

The results of ANOVA and Tukey’s *post hoc* test indicated that BDNF levels of the hippocampus in the EMF group were significantly (*P*<0.05) higher than in the stress group. However, there was no significant difference in BDNF levels of the hippocampus between other groups (Figure 6).

## Discussion

This is the first study investigating the effects of exposure to PS and ELF-EMF on spatial memory, hippocampal BDNF, and serum BDNF and corticosterone levels in adult male offspring. Our results showed that prenatal exposure to stress or ELF-EMF did not significantly affect spatial memory in the adult male offspring. However, the simultaneous induction of stress with the ELF-EMF disrupted the acquisition of spatial memory but did not affect the spatial memory recall phase. 

Some studies have shown the disturbing effect of prenatal exposure to stress. Induction of unpredictable, foot shock stress per day to mothers between the 13^th ^and 19^th ^days of pregnancy reduced the spatial memory of offspring in a MWM task (8). Also, applying ninety minutes of restraining stress between the 17^th ^and 20^th^ days of pregnancy impaired the learning of male and female offspring in the Y and T-shaped mazes; however, shorter periods of restraint or other forms of maternal stress did not impair the acquisition of spatial learning in males or females (9). In addition, the stress of restraining pregnant mothers for 30 min impaired the spatial memory of males, but it did not affect female offspring (30). High maternal corticosterone levels can affect fetal planning (30), and exposure to high levels of glucocorticoids during pregnancy increases the sensitivity of the HPA axis to stress in adulthood (31). Stress affects hippocampal morphology, including glucocorticoid receptor levels, neurogenesis, and dendritic length, and alters overall hippocampal volume (10, 32-34). However, in our study, female rats were exposed to stress before and during pregnancy, which might have caused a compensatory mechanism for adaptation to stress during pregnancy, alleviating the effect of stress exposure on offspring. In addition, depending on the type of stress, age, sex, genetic characteristics of the animal receiving the stress, and several other factors such as the crowded or secluded living environment, the hour of the day when stress is applied, stress may have different effects on the performance and learning of offspring (35-37). 

Induction of ELF-EMF before and during pregnancy did not affect the spatial memory of adult male offspring in this study. Some epidemiological studies have shown that the destructive effects of ELF-MF rise with increasing intensity and exposure time (38-40). In one study, prolonged exposure (12 weeks) to ELF-MF (100 μT and 50 Hz) did not affect the learning ability and spatial memory of healthy adult male rats and Alzheimer’s animals (41). In addition, short-term exposure of mice to ELF-MF (1.1 mT; 50 Hz) did not affect animal performance in the Y maze; however, long-term exposure to 2 mT; 50 Hz ELF-MF reduced recognition of the novel arm (42). The results of some other studies have shown that widespread exposure to ELF-EMF can cause long-term impairments in the learning (43) and spatial memory (44) of immature mice. Contrary to the mentioned studies, the positive effects of exposure to ELF-EMF have also been identified in some studies. In one study, ELF-EMF exposure did not affect the spatial memory of control rats but improved spatial memory and neurogenesis in Alzheimer’s rats (45, 46). 

Moreover, exposure to ELF-EMF increased the potential of the hippocampus, thereby increasing the efficiency of excitatory synapses and improving memory (47). Therefore, the effects of ELF-EMF can be multifactorial and dependent on physical variables such as frequency, polarization, wave properties, duration and direction of exposure to the waves, dielectric properties, conductivity, and water volume of tissues, and environmental factors such as humidity, temperature, and biological variables such as species, body shape and size, weight, body geometry, nutritional status, and health status (40). In addition, in the present study, female rats were exposed to ELF-EMF before and during pregnancy. This might have caused a compensatory mechanism for adaptation to the electromagnetic field during pregnancy, lightening its effect on the offspring.

The present study also directly investigated the effect of simultaneous prenatal exposure to stress and ELF-EMF on a recall-like task (spatial memory). Spatial memory tasks have much in common with cued recall tasks, and the rat, based on distal extra maze cues, must remember the location of a hidden target (48). Combined induction of stress with the ELF-EMF disrupted the acquisition of spatial memory but did not affect the spatial memory recall phase. This finding might bring up another type of acquisition than extra maze cue-based acquisition or a longer acquisition phase to recall the platform’s location during the probe test in these animals. In summary, our findings provide strong support for the conclusion that exposure to either stress or ELF-EMF does not affect spatial memory in adult male offspring. These results suggest an essential reason that some earlier reports have not found that exposure to stress before pregnancy might cause some compensatory mechanisms that decrease the effect of stress during pregnancy on the offspring. This also probably confirms that hippocampal damage may not have been sufficiently complete to reveal a deficit. However, further studies on the number of corticosterone receptors in the hippocampus, the size of the hippocampus, and levels of growth factors are needed to understand the exact mechanisms. 

In our study, PS significantly raised serum corticosterone levels in the adult male offspring. However, PS caused an insignificant decrease in the serum and hippocampal BDNF levels. Additionally, serum corticosterone concentration was significantly lower in the ELF-EMF group, and serum and hippocampal BDNF levels were significantly higher than in the stress group. However, concomitant exposure to stress and ELF-EMF showed a decrease in the serum corticosterone levels and increases in the serum and hippocampal BDNF levels compared to the stress group, although these changes were insignificant. 

Consistent with our research, one study showed that maternal exposure to stress during pregnancy increased serum corticosterone levels and lowered BDNF levels in the brain of rodent offspring (49). Stress was also found to decrease the level of BDNF in the hippocampus of adult male Wistar rats (50). Some other studies have also shown the inverse correlation between serum corticosterone levels and BDNF (51, 52). Activation of the HPA axis could affect BDNF signaling (53), and the levels of BDNF in the hippocampus are decreased under the influence of many stressors (54). BDNF influences neuronal development, differentiation, maturation, and activity in the hippocampus (55). Studies in animal models indicate chronic stress increases neuronal apoptosis via up-regulation of caspase activity and downregulation of neurotrophic factors such as BDNF (50, 56, 57). In the current study, corticosterone and BDNF measurements were performed on the 90^th^ PND, and the impact of PS on BDNF was insignificant. The effect of PS on the offspring may decrease as the age increases. Consistent with our study, one study measured hippocampal BDNF levels at 90th PND, and no differences in BDNF levels were found in the hippocampus or amygdala in the stressed group (58). However, another study on the effect of PS at 42nd PND showed impaired memory and attenuated hippocampal BDNF levels (59). Another study indicated that PS decreased  BDNF expression in the hippocampus of male offspring at PND 21 and 80; however, this effect was more significant at the 21^st^ than in the 80^th^ PND (60). In addition, in our previous study, we showed that the impact of PS was much more apparent in female offspring at the 42^nd^ PND (14). Depending on the type of stress, age, sex, and the hour of the day when stress is applied, stress may have different effects (35, 61, 62). A few studies have concluded that the effects of PS are gender dependent. Given the impact of gender on stress-related behaviors (63), it is safe to assume that the effects caused by PS would be sexually dimorphic (64-66). PS exposure impacts adolescent development of the prefrontal cortex in males but not females, whereas changes in the PFC in females do not emerge until adulthood (67).

Our results also showed that ELF-EMF induction did not change the serum corticosterone levels compared to the control group; however, it decreased serum corticosterone levels compared to the stress group. Exposure to ELF-EMF increased plasma corticosterone, the stress hormone, concentration in one study (68). However, acute exposure to ELF-EMF in another study did not increase corticosterone (69). The effects of ELF-EMF can be dependent on physical variables such as frequency, polarization, duration, and direction of exposure to the waves (70), dielectric properties, conductivity and water volume of tissues, and environmental factors such as humidity, temperature, and biological variables such as species, body shape and size, weight, body geometry, nutritional status, and health status (40). 

Prenatal exposure to ELF-EMF in the current study did not significantly affect the serum and hippocampal BDNF levels; however, serum and hippocampal BDNF levels in the ELF-EMF group were significantly higher than in the stress group. In addition, the pattern of serum BDNF changes was similar to the hippocampal BDNF alterations, consistent with findings of another study on the positive correlation of blood and hippocampal BDNF levels in rats and pigs (71). In one study, ELF-EMF radiation for 30 days substantially decreased hippocampal BDNF levels in adult male rats (50). Another study indicated that two months of daily pulsed ELF-EMF treatment following a spinal cord injury increased BDNF levels and axonal survival at the lesion area (21). It has also been shown that ELF-EMF exposure (50 Hz; 1mT) can cause an increase in *in vivo* hippocampal neurogenesis (72). Furthermore, EMF exposure can promote cell survival and suppress neuronal apoptosis in the nervous system (73). EMF may cause structural and functional changes in the developing brain and, as a result, behavioral changes (74). Studies examining the effects of EMF exposure during prenatal, postnatal, and adolescent periods have shown that neurodevelopment and behavior in children are adversely affected (75, 76). 

In addition, findings of the current study showed that in the 90^th^ PND of male offspring, simultaneous prenatal exposure to stress and ELF-EMF reduced serum corticosterone levels and increased serum and hippocampal BDNF levels compared to the stress group, although these changes were insignificant. Few studies have been conducted on the effects of simultaneous prenatal exposure to stress and ELF-EMF. Our previous study indicated PS and ELF-EMF augmented anxiety-like behavior induced by maternal stress. In addition, accompanying PS and ELF-EMF exposure significantly decreased corticosterone levels in the S-EMF group compared to the stress group in female offspring (14). In a recent study, PS and ELF-EMF showed a dramatic decrease in BDNF in female offspring at the 42^nd^ PND. This study claimed that PS and ELF-EMF during pregnancy could lead to significant neurodegeneration in the hippocampus and prefrontal cortex, resulting in anxiety-like behavior in female offspring (24). This study’s findings on BDNF changes are inconsistent with our results, which might arise from the age or sex of the animals in these two studies. Moreover, in another study, which was directed at adult male Wistar rats, increased values of plasma corticosterone were found in restraint stress and ELF-EMF exposed groups; this effect was more evident in the RS + ELF-EMF group. They suggested that chronic exposure to ELF-EMF is similar to physiological stress and induces changes in brain lipid profile (68). 

**Table 1:** Chronic mild stress schedule for female rats to induce stress before and during pregnancy

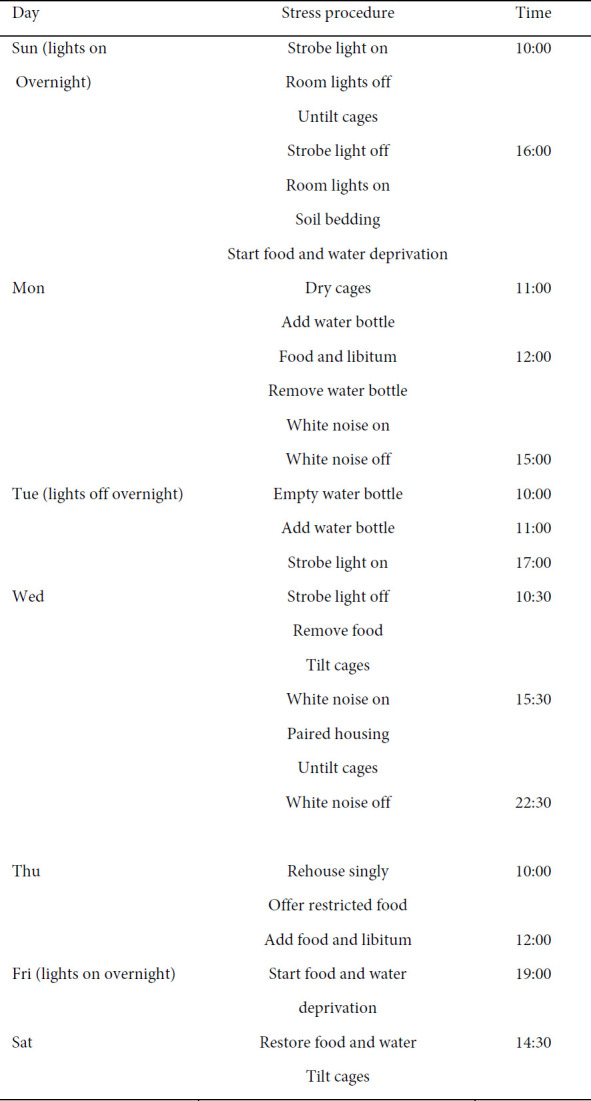

**Table 2:** Effects of prenatal stress and Extremely low-frequency electromagnetic field; (ELF-EMF) exposure on body and brain weight in male adult rat offspring

Groups	Control	Stress	EMF	S+EMF
Parameter				
Body weight (gr)	285.71± 4.96	276.43± 10.15	300.14± 17.95	272.86± 11.71
Brain weight (gr)	1.46± 0.062	1.37 ± 0.016	1.37± 0.04	1.38± 0.0156

Results are displayed as SEM ± Mean (n=8). * Indicates *P*<0.05, ** indicates *P*<0.01, and *** indicates *P*<0.001 in comparison between B1 and B2 within groups.

ELF-EMF: Extremely low-frequency electromagnetic field; MWM: Morris Water Maze

Results are displayed as Mean ± SEM (n=8). ** indicates *P*<0.01, and *** indicates *P*<0.001 compared to the control group.

ELF-EMF: Extremely low-frequency electromagnetic field; MWM: Morris Water Maze

Results are shown as Mean ± SEM (n=8). * Indicates *P*<0.05 com-pared to the control group, and # Indicates *P*<0.05 compared to the stress group

EMF: Electromag-netic field; S: Stress; ELF-EMF: Extremely low-frequency electromagnetic field; BDNF: Brain-derived neurotrophic factor

A representative immunoblotting image of BDNF in different groups (A). The graph shows the protein expression of hippocampal BDNF (B). Data represent the mean ± SEM for six animals in each group. # Indicates *P*<0.05 compared to the stress group

EMF: Electromagnetic Field; S: Stress; ELF-EMF: Extremely low-frequency electromagnetic field; BDNF: Brain-derived neurotrophic factor

## Conclusion

PS or ELF-EMF exposure did not affect spatial memory in the male adult offspring. However, simultaneous prenatal exposure to stress and ELF-EMF impaired their spatial memory acquisition phase. Moreover, ELF-EMF increased serum and hippocampal BDNF levels and decreased serum corticosterone levels compared to the stress group. This highlights the intricate relationship between these factors and brain development later in life. Further studies are needed to fully determine the exact mechanisms and long-term effects of PS and ELF-EMF exposure on memory and hippocampal changes in offspring based on gender and age. 
